# A chromosome-scale genome assembly for Clark’s Nutcracker (*Nucifraga columbiana*)

**DOI:** 10.1093/g3journal/jkag055

**Published:** 2026-03-04

**Authors:** Peter Innes, Matthew D Carling, Ethan B Linck

**Affiliations:** Department of Ecology and Evolutionary Biology, University of Colorado, Boulder, CO 80309, United States; Department of Zoology and Physiology, University of Wyoming, Laramie, WY 82071, United States; Department of Ecology, Montana State University, Bozeman, MT 59717, United States

**Keywords:** Clark’s Nutcracker, Whitebark Pine, *Pinus albicaulis*, Corvidae, PSMC, genome assembly

## Abstract

Focal and intelligent passerine songbirds, corvids (Aves: Corvidae) have served as models for research on the genomics of hybridization and cognition. Clark’s Nutcracker (*Nucifraga columbiana*), the sole North American member of its genus, is distributed in mountain forests from northern Mexico to northern British Columbia. A seed predator, *N. columbiana* is highly reliant on pine nuts from *Pinus* spp. conifers, which it both consumes directly from cones and caches for future use. Since cached seeds may be forgotten or not needed, it is a major seed disperser for high elevation pines—in particular Whitebark Pine *P. albicaulis*. Previous studies of genetic variation in Clark’s Nutcracker found range-wide panmixia and generally high levels of heterozygosity at a handful of nuclear and mitochondrial loci, potentially due to seasonal elevational movements and long-term dispersal. An earlier genome assembly from low-coverage short-read data was highly fragmented and has not to date been used as the basis for population-level resequencing. Here, we report on the first chromosome-scale genome assembly for *N. colombiana*. We generated long-read sequencing and genome conformation mapping data from tissues sampled from a male *N. columbiana* individual in Wyoming, USA. These data were assembled into a highly contiguous and complete assembly, which showed strong chromosomal synteny with New Caledonian crow (*Corvus moneduloides*). We also found evidence of a long-term decline in effective population size dating back to the Pleistocene, after accounting for a technical artifact common to demographic inference using the pairwise sequential Markovian coalescent. These findings raise concerns about the future viability of the species and its mutualist *P. albicaulis*; we hope the assembly will motivate further comparative and conservation genomics research.

## Introduction

Corvids (Aves: Corvidae) are a cosmopolitan clade of passerine songbirds that includes crows and ravens, jays, and magpies. Focal and known for their intelligence and complex social and behavioral phenotypes, multiple species in the clade have served as models for research on the genomics of speciation and hybridization (Poelstra et al. 2014; Vijay et al. 2016; Knief et al. 2019; Slager et al. 2020; Metzler et al. 2021), and cognition and tool use (Sutton et al. 2018; Dussex et al. 2021). Sister to the species-rich genus *Corvus* and their close relatives jackdaws (*Coloeus* spp.), *Nucifraga* nutcrackers are a clade of four species with largely Holarctic distributions and similar seed-predator ecologies that diverged from their closest relatives at the start of the Pliocene (c.a. 5 ma) (McCullough et al. 2023). Prone to irruptive dispersal in search of ephemeral food resources (ie conifer masting events), members of the genus appear to show limited geographic differentiation (Dohms and Burg 2013, 2014; de Raad et al. 2022).

Clark’s Nutcracker (*Nucifraga columbiana*), the sole North American member of its genus, is distributed from northern Mexico to northern British Columbia, and from the Coast Range of California to the Front Range of Colorado. Though their diet varies regionally, it is dominated by *Pinus* spp. pine nuts, augmented by the seeds of other conifers (such Douglas Fir *Pseudotsuga menziesii*), pollen, insects, and small vertebrates (Schaming et al. 2024). *Nucifraga columbiana* both consumes seeds directly and caches them for future use. Because cached seeds may be forgotten or abandoned, it is thus a major seed disperser for high elevation pines—particularly the five-needled Whitebark (*P. albicaulis*) and Limber (*P. flexilis*) Pines. Where the two species co-occur, the tightness of their association and Whitebark Pine’s adaptations to dispersal by *N. columbiana* has been widely described as a mutualism (Hutchins and Lanner 1982; Tomback 1982). This hypothesis and its ramifications for conservation in the face of climate change- and disease-driven declines in *P. albicaulis* have led to decades of research on diverse aspects of the behavior and evolutionary ecology of *N. columbiana* (Tomback 1982; Bednekoff and Balda 1996; Siepielski and Benkman 2007; Lorenz et al. 2011; Schaming 2016).

Despite well-developed genomic resources for other corvids (five *Corvus* spp. and one *Aphelecoma* sp. reference genomes were available on NCBI’s Genome Data Viewer as of October 22nd, 2025; Rangwala et al. 2021), *Nucifraga* nutcrackers have seen limited sequencing effort and been the focus of few population genetics studies to date. Multilocus Sanger sequencing data indicates an absence of geographic structure but substantial genetic variation (measured by observed heterozygosity $H_{o}$ and haplotype diversity) in *N. columbiana* and what was formerly referred to as the Eurasian Nutcracker *N. caryocatactes* (Dohms and Burg 2013, 2014). A highly fragmented, short-read whole genome sequence for Clark’s Nutcracker was included as part of a broader proof-of-concept of the utility of low coverage reference genomes but included little biological interpretation of observed genetic variation, which likely included artifacts from constrained sequencing effort (Card et al. 2014). More recently, a study of the population genomics of *N. cayocatactes* across its range found support for three distinct, genetically isolated lineages within Eurasian Nutcracker (de Raad et al. 2022), leading to the elevation of Southern Nutcracker *N. hemispila* (itself containing four subspecies) and Kashmir Nutcracker *N. multipunctata* to species status (Gill et al. 2025). Despite distinct conifer mutualisms across lineages, the observed timing and pattern of divergence appeared similar to other, codistributed passerines; the authors thus downplayed local adaptation as a driver of speciation in favor of traditional Pleistocene biogeographic factors.

To aid research on corvid comparative genomics and the evolutionary ecology and conservation of *N. columbiana*, we generated a haplotype-resolved, chromosome-scale genome assembly using long-read sequencing and genome conformation mapping data from tissues sampled from a male individual collected in Wyoming, USA. Here, we introduce the resource, report on technical aspects of the assembly and observed heterozygosity, and explore patterns of chromosomal synteny with New Caledonian crow *Corvus moneduloides*. We further infer demographic history from our assembly using a Pairwise Sequential Markovian Coalescent (PSMC) approach.

## Materials and methods

### Specimen collection and tissue sampling

We sampled heart, liver, and pectoral muscle tissues from an adult male Clark’s Nutcracker collected on 2023 October 2nd in Medicine Bow National Forest, Albany Co., Wyoming, USA (41.239667, −105.450667). All tissues were flash frozen in liquid nitrogen in the field. Collecting was permitted by Wyoming Game & Fish Permit (#33–754), the US Fish & Wildlife Service (Permit #MB06336A), and University of Wyoming IACUC #2022–0142. The individual was prepared as a voucher specimen (UWYMV Tissue: B-3379); it is pending accession into the University of Wyoming Museum of Vertebrates (UWYMV) ornithology collections.

### DNA extraction and sequencing

Approximately 1 g of pectoral muscle was sent to Dovetail Genomics (Cantata Bio, LLC), where company staff isolated high molecular weight DNA using a Qiagen Genomic-tip procedure. Briefly, 100 mg of muscle tissue was first incubated with 19 μL RNase and 500 19 μL protease in G2 lysis buffer at 50 ∘C for 2 h. A 10 μL of this solution was taken for total mass determination, which indicated a concentration of 1.76 μg/mL. After purification and elution with a MINI column, the sample was precipitated using isopropanol, resulting in spool formation; it was then transferred to 100 μL Tris-EDTA buffer and incubated overnight at 50 ∘C.

The DNA sample was quantified using a Qubit 4.0 Fluorometer (Life Technologies, Carlsbad, CA, USA), and its size distribution was assessed with a Femto Pulse instrument (Agilent Technologies, Santa Clara, CA, USA). The DNA was then sheared on a MegaRuptor 3 (Diagenode, Denville, NJ, USA) to an average size of 15 to 18 kb and subsequently prepared using the SMRTbell Prep Kit 3.0 (Pacific Biosciences, Menlo Park, CA, USA) following manufacturer’s instructions. The sequencing library was size-selected on a BluePippin instrument (Sage Science, Beverly, MA, USA) to remove fragments smaller than 10 to 15 kb, bound to polymerase with the Revio polymerase kit, and sequenced in one run with a Revio sequencing plate and a Revio SMRT Cell Tray on the PacBio Revio instrument.

From the same muscle tissue, DNA for chromatin conformation capture sequencing was first fixed in place in the nucleus with formaldehyde and then extracted as described above. Fixed chromatin was digested with DNAse I, and chromatin ends were repaired and ligated to a biotinylated bridge adapter followed by proximity ligation of adapter-containing ends. After proximity ligation, crosslinks were reversed and the DNA was purified. Purified DNA was treated to remove biotin that was not internal to ligated fragments. This Dovetail Omni-C sequencing library was completed with the addition of NEBNext Ultraenzymes (New England Biolabs, Ipswich, MA, USA) and Illumina-compatible adapters. Biotin-containing fragments were isolated using streptavidin beads before PCR enrichment of the library. It was then sequenced on an Illumina NovaSeq X Plus machine with 150 bp paired-end reads.

### Genome assembly

Prior to assembly, we removed PacBio HiFi reads with remnant adapter sequence using hifiadapterfilt (Sim et al. 2022), although this was probably not necessary as only 58 reads (0.00086% of total) were flagged and removed. We also estimated genome size, heterozygosity, and repetitiveness using k-mer frequency profiling of the raw HiFi data, implemented in GenomeScope2 (Ranallo-Benavidez et al. 2020), with the settings −l 40 −k 21 −p 2.

We assembled haplotype-phased contigs with hifiasm [v0.23.0 Cheng et al. 2021] using the HiFi and Omni-C reads and default settings. We ran the FCS-GX decontamination tool (Astashyn et al. 2024) to remove foreign contaminating sequences before scaffolding. This tool uses a custom database of NCBI RefSeq sequences and GenBank assemblies totaling ∼700 Gbp and representing over 48,000 taxa with emphasis on prokaryotes, viruses, and eukaryotes with small genomes that are commonly found as contaminants. For hap1, 341 sequences totaling 11,798,270 bp were excluded; for hap2, 9 sequences totaling 426,226 bp were excluded. Nearly, all contaminant sequences (333) were mosses; seven were identified as bacteria, and there was one insect sequence. We aligned the Omni-C data to the contigs of both haplotypes and processed alignments following the Dovetail Genomics alignment pipeline (https://dovetail-analysis.readthedocs.io/en/latest/). We then used YAHS (Zhou et al. 2023) to scaffold the contigs.

We visualized Omni-C contact maps using PretextMap and PretextView and performed manual curation of both haplotypes following the strategy outlined previously (Howe et al. 2021). Overall, minimal curation was required. For hap1, we made 3 cuts in contigs, 0 breaks at gaps and 7 joins; for hap2, we made 0 cuts in contigs, 0 breaks at gaps and 6 joins.

We calculated basic assembly metrics with gfastats (Formenti et al. 2022). We assessed the completeness of the curated hap1 and hap2 assemblies using compleasm (Huang and Li 2023), which is a reimplementation of BUSCO (Benchmarking Universal Single-Copy Orthologs), and the passeriformes_odb12 dataset, which comprises 6,684 orthologs. We visualized chromosomal colinearity (ie synteny) of both assemblies compared to New Caledonian Crow reference genome (Rhie et al. 2021; NCBI GenBank accession GCA_009650955.1) using JupiterPlot (Chu 2018), limiting the number of visualized nutcracker scaffolds to those making up 99% of the assembly. We chose the New Caledonian Crow genome for synteny analysis because of its high quality and use in previous Corvidae genome comparisons (Benham et al. 2023; DeRaad et al. 2023). This reference genome was also used to make a preliminary annotation of our curated hap1 assembly via liftoff (Shumate and Salzberg 2021), implemented with default settings.

We assembled a mitochondrial genome sequence with MitoHiFi (Uliano-Silva et al. 2023) using the assembled scaffolds as input and a previously published Clark’s nutcracker mitogenome sequence as a reference (NCBI GenBank accession NC_022839.1; Barker et al. 2015). Only the hap1 assembly was found to contain mitochondrial scaffolds, which we removed to avoid redundancy with the complete mitochondrial genome. Software information and version numbers for the full assembly pipeline are provided in Table 1.

**Table 1.** Genome assembly pipeline.

Assembly step	Software	Version
Raw data filtering	HiFiAdapterFilt	3.0.0
k-mer counting	Meryl (k = 21)	1.4.1
Genome profiling	GenomeScope (−l 40 −k 21 −p 2)	2.0.1
De novo assembly	Hifiasm (Hi-C mode)	0.23.0
Contamination screening	FCS-GX	0.5.5
Alignment of Omni-C data	bwa mem (−5SP −T0)	0.7.17
Processing of Omni-C alignment	pairtools (Dovetail pipeline)	1.1.2
Scaffolding	YAHS	1.2.2
Contact map generation	PretextMap	0.1.9
Contact map manual curation	PretextView	1.0.3
Contact map visualization	PretextSnapshot	0.0.4
Assembly metrics	gfastats	1.3.9
Assembly BUSCO completeness	Compleasm (−l passeriformes_odb12)	0.2.7
Synteny visualization	JupiterPlot (ng = 99 m = 1,000,000)	1.1
Gene annotation	Liftoff	1.5.1
Mitogenome assembly	MitoHiFi (−o 2)	3.2.3

### Demographic inference

We inferred the population size history of Clark’s Nutcracker using the PSMC (Li and Durbin 2011). We aligned the PacBio HiFi reads to the completed hap1 assembly with minimap2 and used bcftools mpileup and bcftools call (Danecek et al. 2021) to generate a diploid consensus sequence, which was subsequently filtered for minimum and maximum read depth of 24 and 146—a third and twice the average, respectively—following PSMC recommendations. A previous study found that PSMC parameters −N30 −t5 −r5 −p “4+30*2+4+6+10” were suitable for diverse avian species (Nadachowska-Brzyska et al. 2015). We followed this guidance but additionally tuned the −p setting, which controls the atomic time intervals and free interval parameters. We split the first time interval in order to test for a known technical artifact identified in numerous species: a sharp population size peak immediately preceding a recent population decline (Hilgers et al. 2025). Specifically, we ran PSMC three times with different −p settings: (i) 4+30*2+4+6+10, (ii) 2+2+30*2+4+6+10, and (iii) 1+1+1+1+30*2+4+6+10. One-hundred bootstrap replicates were performed for each analysis. Mutation rate and generation time were set to $0.3\times 10^{-8}$ (Vijay et al. 2016) and 7 yrs following a previous nutcracker genomic study (de Raad et al. 2022).

## Results and discussion

**Fig. 1.**
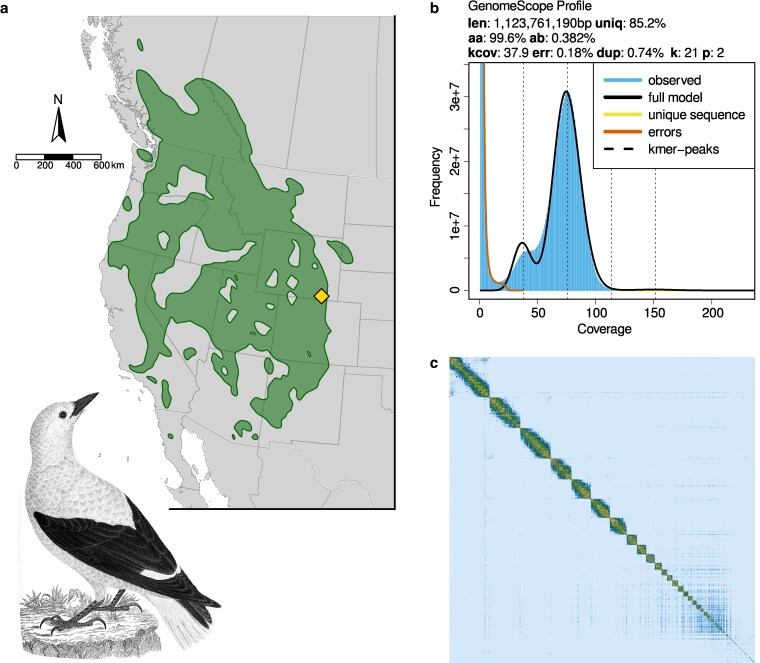
a) Map of the approximate year-round distribution of Clark’s Nutcracker. The sequenced bird was collected at the location marked with a yellow diamond. Distribution data were obtained from eBird (Fink et al. 2025). Illustration is by Alexander Wilson and is in the public domain in the United States. b) K-mer genome profiling using PacBio HiFi reads and GenomeScope2. The main peak captures average k-mer coverage of homozygous base pairs while the shorter peak (kcov = 37.9) represents that of heterozygous base pairs. Other estimated metrics are: total estimated genome length (len), percentage of genome containing nonrepetitive sequence (uniq), overall rate of homozygosity (aa), overall rate of heterozygosity (ab), sequencing error rate (err), and average rate of read duplication (dup); “k” and “p” refer to settings for k-mer length and ploidy. c) Omni-C contact map for hap1 depicting a high-quality chromosome-scale assembly. The diagonal line represents the linear sequence of the assembly, and the image is a symmetric matrix. Frequency of contact among genomic regions, which corresponds to spatial proximity, is represented by signal intensity ranging from low (blue/lightly shaded regions) to high (yellow and red/more darkly shaded regions).

Our sequencing efforts produced 88.6 Gb of HiFi sequence data and 65.9 Gb of Omni-C sequence data. K-mer profiling of the HiFi data showed an estimated genome size of 1.12 Gb, with a heterozygosity of 0.38% and 15% repetitive sequence content (Fig. 1a).

With these data, we assembled a chromosome-scale and haplotype-resolved genome sequence for Clark’s Nutcracker. Haplotype 1 comprises 806 scaffolds totaling 1.215 Gb with a contig N50 of 13.01 Mb, and haplotype 2 has similarly high contiguity (Table 2). A contact map for hap1, representing the chromosome-scale quality of the assembly, is visualized in Fig. 1b. Both assemblies contained complete and single-copy orthologs for 99.5% of the 6,684 Passeriformes BUSCO markers, suggesting highly complete assemblies (Table 2). Homology-based genome annotation relying on a closely related corvid species, the New Caledonian Crow, resulted in 20,306 of 20,680 gene features (98.2%) successfully mapped. These contiguity and completeness metrics are among the highest for existing chromosome-scale genome assemblies in the Corvidae (Benham et al. 2023). The mitochondrial genome assembly totaled 16,896 bp, which is similar to existing *N. columbiana* and Eurasian Nutcracker *N. caryocatactes* mtDNA assemblies (16,905 and 16,914 bp, respectively; Barker et al. 2015; Meng et al. 2020).

**Table 2.** Genome assembly quality metrics.

Assembly metric	hap1	hap2
Number of scaffolds	806	872
Total scaffold length (bp)	1,215,271,717	1,302,111,241
Scaffold N50 (bp)	76,143,722	66,284,030
Scaffold L50	6	7
Largest scaffold (bp)	160,630,474	159,758,688
Smallest scaffold (bp)	1,000	1,148
Number of gaps in scaffolds	260	276
Number of contigs	1,066	1,148
Contig N50 (bp)	13,009,141	12,429,319
Contig L50	26	32
GC content	43.75%	44.40%
BUSCOs^a^	S 99.48%; D 0.24%; F 0%; M 0.28%	S 99.51%; D 0.48%; F 0%; M 0.01%

^a^passeriformes_odb12; n = 6,684.

**Fig. 2.**
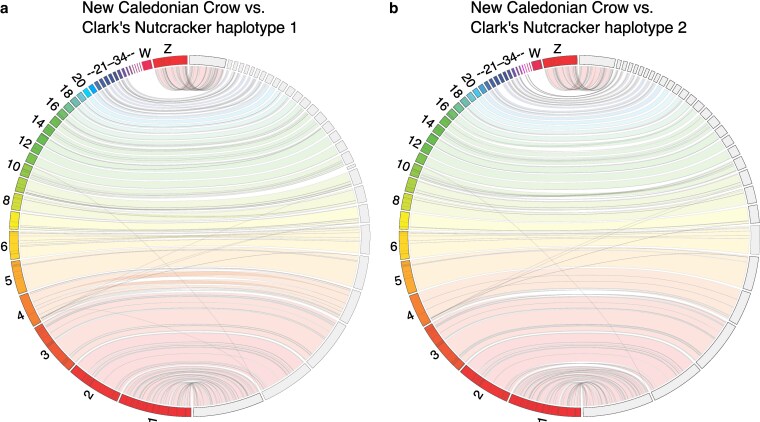
Chromosomal synteny between New Caledonian Crow and Clark’s Nutcracker, visualized with JupiterPlot. In both panels, New Caledonian Crow chromosomes are on the left hemisphere and Clark’s Nutcracker chromosomes are on the right. Colored bands indicate sequence collinearity between assemblies. The lack of homology with the New Caledonian Crow W chromosome is because a male bird was sequenced.

As expected for closely related bird species, chromosomal synteny between Clark’s Nutcracker and the New Caledonian Crow was generally high and lacked large structural rearrangements (Fig. 2). The largest gap in collinearity between assemblies occurred at the New Caledonian Crow’s W sex chromosome, corroborating notes from specimen preparation that our sequenced Clark’s Nutcracker was male, the homogametic sex (ZZ).

**Fig. 3.**
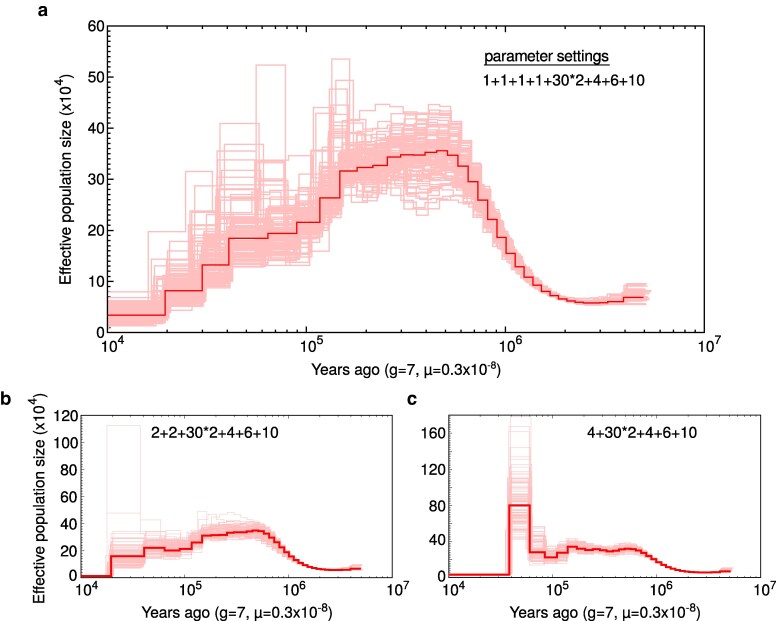
Clark’s Nutcracker population size history inferred with the PSMC. The panels show the sensitivity of inference to different time interval parameter settings. a) The first four atomic time intervals are split into four separate windows (1+1+1+1). b) The first four intervals are split into two windows (2+2). c) The first four intervals are contained in a single window as in PSMC default settings, resulting in a false peak followed by a sharp decline.

Inference of historical effective population size ($N_{e}$) with PSMC suggested that Clark’s Nutcracker has experienced a long-term population decline beginning in the late Pleistocene, approximately 500 kya (Fig. 3a). We obtained this result after finding that population size history was sensitive to time interval parameter settings in a way that resembled a known technical artifact producing false population size peaks (Hilgers et al. 2025). Specifically, when the first atomic time window was kept at the default value of 4, we observed a large population size peak followed immediately by a recent decline (Fig. 3c). Splitting the first window from 4 to 2+2 removed this peak, with the exception of two outlier bootstrap replicates (Fig. 3b). Splitting the first window again (to 1+1+1+1) appeared to largely resolve the issue and revealed a consistent decline beginning ∼500 kya (Fig. 3a). These results emphasize previous recommendations to test PSMC parameter settings beyond the default, particularly for the initial time window (Hilgers et al. 2025).

Earlier work on the demographic history of Eurasian nutcrackers inferred recent $N_{e}$ peaks followed by steep declines were inferred for two of three (*N. caryocatactes*) subspecies (*N. c. macrorhynchos* and *N. c. japonica*; de Raad et al. 2022). We raise the possibility that these peaks might be technical artifacts, as they both closely resemble the false-peak pattern demonstrated here and by Hilgers et al. (2025), and as the first time interval setting in the study was kept to the problematic default value of 4. In contrast, the third Eurasian Nutcracker subspecies included in the authors’ analysis—*N. c. caryocatactes*—showed an effective population size history comparable to what we infer for Clark’s Nutcracker (Fig. 3a). Similar long-term declines from a peak in the mid-Pleistocene appear in other bird species of the temperate Northern Hemisphere (eg Bald Eagle, Rock Dove, and Turkey Vulture; Nadachowska-Brzyska et al. 2015) and can likely be explained by dramatic shifts in climate and vegetation.

Our analysis suggests that by c.a. 10,000 yr ago—roughly the end of the Last Glacial Period—the effective population size of Clark’s Nutcracker had dropped below 50,000 (Fig. 3a). Despite an apparent history of decline, this estimate of $N_{e}$ is comparable to or larger than those reported for other bird species in the literature (eg Nadachowska-Brzyska et al. 2015). Consistent with this observation are the results of a previous phylogeographic study of Clark’s nutcracker using mtDNA and microsatellite markers that found high population-level heterozygosity, interpreted by the authors as a sign of large census population sizes and potential panmixia (Dohms and Burg 2013). Though a direct comparison of observed heterozygosity between that study and ours is impossible given differences in marker type and scale, our estimate of $H_{o}$ is notably lower than those from recently published Steller’s Jay (0.64%; Benham et al. 2023) and California Scrub Jay (0.66%; DeRaad et al. 2023) genomes generated with similar methods. This may be in part due to differences in the sex of the sampled individual—both cited studies sequenced heterogametic female birds, while our study sequenced a homogametic male—but the pattern awaits further investigation.

Clark’s Nutcracker has attracted behavioral and ecological study for decades (Tomback 1980, 1982; Bednekoff and Balda 1996; Lorenz et al. 2011; Schaming 2016). Nonetheless, the distribution of genetic diversity in the species remains poorly documented, perhaps due to the assumption that long distance dispersal and attendant gene flow counteracts regional differentiation via genetic drift and is a major force facilitating or hindering local adaptation (Dohms and Burg 2013). We concur that gene flow is likely pervasive across the range of the species but highlight the possibility of as-yet undescribed patterns of population subdivision or isolation-by-distance, particularly if mating is decoupled from irruptive movements. We further emphasize the need for evaluating the impact of ongoing habitat loss (eg climate change-associated mortality in Whitebark Pine) on levels of neutral and functional genetic variation. Genome-wide resequencing data paired with field observations and individual-level GPS or radio telemetry tracks will be needed to make strong inferences about the evolutionary history, present status, and future vulnerability of this charismatic and important bird (Mather et al. 2020).

## Data Availability

Raw sequence data (PacBio HiFi and Illumina Omni-C) and final genome assemblies are publicly available on GenBank (BioSample: SAMN52876107). Scripts used in assembly and quality control are available on GitHub (https://github.com/peterinnes/nucifraga_assembly). Raw data are accessioned under BioProject PRJNA1347870; the hap1 and hap2 assemblies are accessioned under BioProjects PRJNA1416957 and PRJNA1416956, respectively. Our annotation and assemblies of both haplotypes and the mitochondrial genome are available via Figshare (https://doi.org/10.25387/g3.30594014).
